# Current perspectives on video and audio recording inside the surgical operating room: results of a cross-disciplinary survey

**DOI:** 10.1007/s13304-020-00902-7

**Published:** 2020-10-26

**Authors:** Floyd W. van de Graaf, Özgür Eryigit, Johan F. Lange

**Affiliations:** Department of Surgery, Erasmus University Medical Center (Erasmus MC), University Medical Center Rotterdam, P.O. Box 2040, 3000 CA Rotterdam, The Netherlands

**Keywords:** Laparoscopic surgery, Multimedia, Operating room, Video recording, Audio recording

## Abstract

The availability of intraoperative multimedia recording is increasing. Considering the growing call for physicians’ accountability, it is inevitable that multimedia will play an important role in aiding quality control by improving the adequacy of operative reporting. However, the perspectives of medical professionals on this matter are poorly known. In this cross-disciplinary survey, we aimed to investigate the current viewpoints concerning the use of multimedia recording in the operating room. We conducted an electronic survey among all affiliated members of the Association of Surgeons of the Netherlands, the Dutch Urological Association and the Dutch Society of Obstetrics and Gynecology containing questions regarding current use of intraoperative recording and the level of likelihood or objection for certain scenarios. The response rate was 27.8%. The survey encompasses 370 (54.5%) surgeons, 71 (10.5%) urologists, 80 (11.8%) gynecologists, and 158 (23.3%) residents in training. 52.4% of respondents feel that the currently used operative report is insufficient for future quality requirements. 58.5% think it is unlikely they would behave differently during surgery when intra-operative video recording is applied. 82.8% think it is unlikely that their surgical methods would be altered. 63.8% of respondents preferred only video registration when intraoperative recording is implemented. The majority of respondents agree that the current method of operative reporting is insufficient for future quality requirements. There is support for intraoperative video recording, however, legal transparency is needed before either intraoperative video or audio recording could be implemented to protect not only the patients, but also the healthcare providers.

**Electronic supplementary material:**

The online version of this article (10.1007/s13304-020-00902-7) contains supplementary material, which is available to authorized users.

## Introduction

During the last decade, the use of multimedia in the context of the operating room has increased rapidly. Capturing video, still images or sound have become an essential part of daily practice in many surgical disciplines, with the potential to benefit either individual patient care or treatment as a whole. Alongside photo-documentation of laparoscopic female sterilization, probably the best-known example of multimedia documentation is that of the critical view of safety (CVS) on photo or video in laparoscopic cholecystectomy as an auxiliary to the narrative operative report [1]. This approach has become an essential part in laparoscopic cholecystectomy procedures in the Netherlands and is also recommended in the USA [2, 3]. Prior research demonstrated that the traditional narrative operative report does not adequately reflect reality in laparoscopic cholecystectomy [4–6]. One method to ameliorate the accuracy of these reports could be the use of synoptic reporting, utilizing a structured template to construct an operative report, diminishing the amount of data omitted and effectively increasing its integrity [7–13]. Utilizing intraoperative video recording in synergy with a written operative report also proved to be feasible and furthermore, superior to the classic narrative operative report alone [5, 6]. For an even better understanding of the operative procedure upon review, simultaneous audio recordings could be a valuable option to further reduce discrepancies between video recording and operative report [14].

In addition to a boost in reporting quality, use of multimedia documentation could also be invaluable for other purposes, for instance, in the case of surgical quality control and quality assurance. In a study by the Michigan Bariatric Surgery Collaborative, peer-rating of procedural videos of laparoscopic gastric bypass surgery was performed to assess participating bariatric surgeons’ technical skills [15]. The authors reported a relationship between the technical skill quantified on video and postoperative outcomes, confirming that greater technical skill does indeed result in significantly fewer postoperative complications. Taking it a step further, Toronto-based surgeon Dr. Teodor Grantcharov developed the surgical ‘black box’. This recording device, much like its equivalent in aviation, registers data regarding the surgical procedure in real time from multiple inputs, i.e. sound (speech of surgeons and operating room personnel), videos from several angles (surgical site and surrounding areas in the operating room), and patients’ vital signs from the anesthesia workstation, in order to discern the origins of adverse events.[16].

Considering the growing call for physicians’ accountability, it is inevitable that multimedia will play an important role in the foreseeable future and that it will indeed contribute to quality of care. Nonetheless, the views of key players are of great importance in this evolution, and the perspectives of medical professionals in the current surgical climate are poorly known. Therefore, in this cross-disciplinary survey, we aimed to investigate the current viewpoints of surgical specialists and residents in training concerning the use of multimedia recording in the operating room. Inquiries were made regarding their current practice in documenting surgical procedures, their views in regard to the added value and the exact composition of multimedia recordings, and their perspective on possible privacy and legislative issues in this context.

## Methods

On 20 December 2018, members affiliated to the Association of Surgeons of the Netherlands, the Dutch Urological Association and the Dutch Society of Obstetrics and Gynecology were approached by e-mail to engage in a web-based survey (LimeSurvey, LimeSurvey GmbH. Hamburg, Germany). Respondents not wanting to participate in the survey were provided with an opt-out option. Three reminders were sent to non-responders after initial invitation, with an interval of 4 weeks. Retired surgeons, urologists or gynecologists, approached persons with other functions than surgeon, urologist, gynecologist or resident of one of the corresponding disciplines, and partial responses were excluded from analysis.

### Questionnaire design

This questionnaire consists of 16 questions. Questions 1 through 4 covered respondents’ demographics. Questions 5 through 9 were multiple choice questions regarding the current use of operative reporting and its serviceability in the future, when developments in technology and an increased call for accountability will likely add to the requirements in medical reporting [17]. Questions 10 through 16 were five-point Likert type scales for likelihood or level of objection concerning the use of multimedia in the operating room. The full survey can be found as online supplementary data.

### Statistical considerations

Data were analyzed with IBM SPSS Statistics for Windows, version 25.0 (IBM Corp. Armonk, NY) and Microsoft Excel (Microsoft Corp., Redmond, WA). Data are presented as numbers and percentages. A *P* value of less than 0.05 was considered statistically significant. Groups were compared using Chi-square test or Fisher’s exact test. When responses of two categories were compared within the same group, McNemar’s test was used.

## Results

Invitations to a total number of 3151 e-mail addresses were sent, of which 3056 were successfully delivered. The overall response rate was 876 (27.8%). Replies of 197 respondents were excluded from this survey [112 (56.9%) retired or other function than surgeon, urologist, gynecologist or resident; 85 (43.1%) partial responses]. After exclusion, a total number of 679 complete questionnaires were analyzed.

Among the respondents, 370 (54.5%) were surgeons, 71 (10.5%) were urologists, 80 (11.8%) were gynecologists, and 158 (23.3) were residents in training of the corresponding disciplines.

Of the respondents, 147 (21.6%) currently practice their trade in university hospitals, whereas 428 (63.0%) and 82 (12.1%) work in general teaching and general non-teaching hospitals, respectively. Respondents’ demographics can be found in Table 1.

**Table 1:** Respondent demographics

	Specialists (*n* = 521)
	*N* (%)
Years practicing	
< 5 years	109 (20.0)
5—10 years	129 (25.1)
10—15 years	102 (18.9)
15—20 years	88 (16.2)
> 20 years	90 (18.9)

	Residents (*n* = 158)
	*N* (%)
Year of training	
Year 1	24 (15.2)
Year 2	19 (12.0)
Year 3	27 (17.1)
Year 4	28 (17.7)
Year 5	37 (23.4)
Year 6	23 (14.6)

### Perspectives on the current operative report

Overall, 356 (52.4%) respondents feel that the currently used narrative operative report—without video and/or sound—is insufficient for future quality requirements [183 (49.5%) surgeons, 47 (58.8%) gynecologists, 41 (57.7%) urologists and 85 (53.8%) residents]. There was no significant difference in responses among specialists and between specialists and residents (*P* = 0.267 and 0.850, respectively), nor between differences in practicing years among specialists (less than 5 years, 5–10 years, 10–15 years, 15–20 years or more than 20 years of work experience) or different years of training among residents (year 1 through 6; *P* = 0.333 and *P* = 0.339, respectively).

### Current use of intraoperative multimedia recording

Table 2 delineates the different techniques which respondents reported to be present in their institution. 630 (92.8%) of respondents reported the use of endoscopic camera recording. Respectively, 179 (26.4%) and 85 (12.5%) of respondents indicated that an external camera to record the surgical site, such as a lamp mounted camera, or a camera dedicated to film the surroundings of the operating room, as is the case with the surgical black box among others, is used. A mobile phone is stated to be used to record intraoperative events by 288 (42.4%).

**Table 2:** Reported techniques used in institutions

	Respondents (*n* = 679)
	*N* (%)*
Endoscopic camera feed	630 (92.8)
External camera filming the surroundings of the operating room	85 (12.5)
External camera dedicated to fil the surgical site (e.g. lamp camera)	179 (26.4)
Surgical black box	25 (3.7)
Mobile phone	288 (42.4)
Sound recorder (microphone)	25 (3.7)
None of the above	23 (3.4)
Other	33 (4.9)

Values represent the number and percentage of respondents answering “yes”

Overall, 621 (91.5%) of respondents stated that routine video recording of conventional procedures was not common practice in their department. For endoscopic procedures, this number was 186 (27.4%). There was no significant difference within departments (*P* = 0.791 and *P* = 0.640 for conventional and endoscopic setting respectively). Data of all separate specialties is delineated in Table 3.

**Table 3:** Routine use of intra-operative video recordings, per department

	Surgery (*n* = 486)	Obstetrics and Gynecology (*n* = 112)	Urology (*n* = 81)		Total (*n* = 679)
	*N* (%)	*N* (%)	*N* (%)	*P* value	*N* (%)
Routine use of video recordings during conventional surgery					
Yes	16 (3.3)	2 (1.8)	1 (1.2)	0.791	19 (2.8)
No	445 (91.6)	103 (92.0)	73 (90.1)		621 (91.5)
Don’t know	10 (2.1)	3 (2.7)	2 (2.5)		15 (2.2)
Missing	15 (3.1)	4 (3.6)	5 (6.2)		24 (3.5)
Routine use of video recordings during endoscopic surgery					
Yes	317 (65.2)	73 (65.2)	47 (58.0)	0.640	437 (64.4)
No	128 (26.3)	32 (28.6)	26 (32.1)		186 (27.4)
Don’t know	24 (4.9)	3 (2.7)	4 (4.9)		31 (4.6)
Missing	17 (3.5)	4 (3.6)	4 (4.9)		25 (3.7)

### Retention period

423 (62.3%) respondents did not know the retention period their institution upholds for video recordings of surgical procedures. Residents know the retention period significantly less often than specialists (120 (75.9%) vs. 303 (58.2%); *P* =  < 0.001). There was no significant difference among specialists (surgeons 217 (58.6) vs. gynecologists 44 (55.0%) vs. urologists 42 (59.2%); *P* = 0.821). Of the respondents who do know the retention period in their institution, 20 (2.9%) reported a retention period of less than 30 days, 109 (16.1%) between 30 and 90 days, 40 (5.9%) 90 days and up to a year and lastly 87 (12.8%) reported a period of more than a year.

### Frequency of intraoperative recording

Overall, the number of respondents answering ‘never’ or ‘almost never’ regarding intraoperative video recording was 130 (19.1%) for endoscopic procedures and 483 (71.1%) for conventional procedures. For specialists only, these numbers were 104 (20.1%) for endoscopic procedures and 421 (81.3%) for conventional procedures. When comparing specialists in terms of experience level, there was no significant difference (*P* = 0.710 and *P* = 0.605 for endoscopic and conventional procedures, respectively). Surgeons significantly more often utilize video recording in open procedures than gynecologists and urologists (*P* = 0.002). There was no significant difference among specialists in regard of work experience; *P* = 0.710 and *P* = 0.605 for endoscopic and conventional, respectively) as well as among residents in terms of year of training (*P* = 0.262 and *P* = 0.420 for endoscopic and conventional, respectively).

### Purposes of video recording

Respondents from the surgical department include video in the patient file significantly less often than those from gynecology or urology (41.4% vs. 55.4% vs 49.4%, respectively; *P* = 0.018). There was no significant difference within departments concerning the recording of video files for quality control purposes, educational purposes, or in the context of proctoring (overall percentage 50.5%, *P* = 0.070; 48.5%, *P* = 0.341; 9.7%, *P* = 0.066, respectively). Respondents from the surgical department record video to provide information for patients and their family or for colleagues significantly less often than those from gynecology or urology (23.9% vs. 33.0% vs. 35.8%, respectively; *P* = 0.021). All purposes for intraoperative video recording reported by respondents are delineated in Table 4.

**Table 4:** Purposes of video recording

	Surgery (*n* = 486)	Obstetrics and Gynecology (*n* = 112)	Urology (*n* = 81)		Total (*n* = 679)
	*N* (%)	*N* (%)	*N* (%)	*P* value	*N* (%)
Addition to patient file	201 (41.4)	62 (55.4)	40 (49.4)	0.018	303 (44.6)
For quality control purposes	232 (47.7)	65 (58.0)	46 (56.8)	0.070	343 (50.5)
For educational purposes	238 (49.0)	48 (42.9)	43 (53.1)	0.341	329 (48.5)
In the context of proctoring	55 (11.3)	5 (4.5)	6 (7.4)	0.066	66 (9.7)
To provide information for patients, family and/or colleagues	116 (23.9)	37 (33.0)	29 (35.8)	0.021	182 (26.8)
Other	32 (6.6)	5 (4.5)	5 (6.2)		42 (6.2)

Values represent the number of respondents selecting the given purposes as a reason for video recording

### Behavior in the operating room

Among all respondents, 397 (58.5%) responded that it would be “unlikely” or “very unlikely” that they would behave differently during surgery when intra-operative video recording is applied. 562 (82.8%) responded that it would be “unlikely” or “very unlikely” that their surgical methods would be altered by the presence of intra-operative video recording. When intra-operative video *and* audio recording would be implemented, respondents reported that they would be significantly more likely to behave differently and/or would alter their surgical methods (reports of “unlikely” or “very unlikely”: 232 (34.2%) *P* < 0.001 and 512 (75.4%) *P* < 0.001, respectively). Responses by residents indicated that they would behave differently in the operating room significantly more likely when intraoperative video recording is applied in comparison to responses by specialists (39.7% vs. 30.2%; *P* = 0.047, respectively). When inquired about the effect of video and audio recording, this significant difference increases to 71.0% vs. 56.5% (*P* = 0.003), respectively. No significant difference among specialists with different experience levels was found regarding the effect on their behavior in the operating room or their surgical methods for both the context of video (*P* = 0.465 and *P* = 0.872, respectively) and video with audio (*P* = 0.734 and *P* = 0.329). Cronbach’s alpha of internal consistency for five-point Likert type scale questions in this section was 0.871.

### Privacy and legal concerns

In the context of the recognizability of the respondent in the situation of intraoperative video recording, 252 (37.1%) of respondents find this either “objectionable” or “very objectionable”. 358 (52.7%) find it either “objectionable” or “very objectionable” to be recorded on intra-operative video in regards of medical liability. Finally, 241 (35.5%) find it either “objectionable” or “very objectionable” to be recorded on intra-operative video in the context of quality of surgical care. Among specialists, there was no significant difference concerning the years of practice in the context of recognizability or medical liability (*P* = 0.589 and *P* = 0.071). In the context of quality of surgical care, there was a significant difference between the different levels of experience (*P* < 0.001), with those who are less experienced (5 years or less) more likely to object compared to those with the most experience (20 years or more; 53 (68.8%) vs 21 (32.3%), respectively). Cronbach’s alpha of internal consistency for these questions was 0.726.

### Added value of intra-operative video and sound recording

409 (60.2%) and 222 (32.7%) respondents recognized the added value of intraoperative video and intraoperative video *with* sound as either “likely” or “very likely”. 602 (88.7%) and 419 (61.7%) deemed this for educational purposes. 302 (44.5) and 148 (21.8%), respectively, found intraoperative video and intraoperative video *with* sound useful in providing information for patients, family and/or colleagues. 411 (60.5%) and 269 (39.6%) saw potential in the use of these respective modalities for quality control purposes. 453 (66.7%) and 312 (45.9%) deemed it likely that intraoperative video and intraoperative video with sound respectively would be an addition in the context of proctoring. Finally, 378 (55.7%) and 282 (41.5%) of respondents found it likely that intraoperative video and intraoperative video with sound could play a supportive role in medicolegal proceedings.

Cronbach’s alpha of internal consistency for these questions was 0.84.

### Preferred recording method for intraoperative registration

Table 5 lists an overview of preferred recording methods. 433 (63.8%) of respondents preferred only video if registration of the surgical procedure was implemented. 144 (21.2%) preferred video *and* audio recording. 84 (12.4%) would rather not have any recording at all. 18 (2.7) did not submit any preference.

**Table 5:** Preferred recording method for intraoperative registration

Statements	All respondents (*n* = 679)
	*N* (%)
Video recordings of the entire surgical procedure	211 (31.1)
Video recordings of only the essential steps of the surgical procedure	222 (32.7)
Video and audio recordings of the entire surgical procedure	77 (11.3)
Video and audio recordings of only the essential steps of the surgical procedure	67 (9.9)
No video and audio recordings	84 (12.4)
No preference	18 (2.7)

## Discussion

An increasing number of studies are exploring the values of multimedia recording in the surgical setting today. Some are exploring its role in surgical quality analysis and control [4–6, 16, 18]. Some assess its part in the amelioration of operative reporting [6, 14]. Others examine its part in surgical education [19–21]. While each an addition to the growing knowledge on this matter, none are currently implemented in a widespread manner. End users, in this case the surgical specialists, have yet to voice themselves regarding their viewpoint in intraoperative video and audio recording. To our knowledge, this study has been the first to do so.

About half of the respondents agree with the statement that the currently used narrative operative report, without the addition of intraoperative video and/or sound, is lacking for future quality requirements. Today, the majority of institutions use either dictation devices, typed reports or modified pre-written concept reports. This method of reporting, however, is subjective by nature and often lacks essential information [4, 14].

As expected, endoscopic procedures are far more often recorded by respondents compared to conventional (“open”) procedures. This is mostly due to the fact that the endoscope’s camera function is essential to conduct minimally invasive surgery. Video recording could then be implemented at the press of a button. Therefore, far less use a different, dedicated modality to record surgical procedures on video, such as a camera mounted to the surgical lamp (26.4%) or a fixed camera in the operating room (12.5%). Often, the quality is lacking, or the operator’s head and body are in its line of sight. Furthermore, for dynamic procedures, such as in orthopedic surgery or vascular surgery, it is virtually impossible to capture the essential moments through this method. Noteworthy is the use of mobile phones to record certain aspects of the surgery; about half of respondents have stated to use their mobile phone. This is probably due to the ease of use and the possibility to use the phone’s video call function to consult colleagues or other specialists.

More than half of respondents did not know the duration of the retention period for intraoperative video recordings in their institution. Most that did know, reported a retention period between 30 and 90 days. Rules regarding the production and handling of medical documentation have been laid down in the Health Insurance Portability and Accountability Act (HIPAA) for the United States and the European General Data Protection Regulation for the European Union [22, 23]. However, a specific time period is stipulated in neither. Instead, referral to local legislation is made.

The results of this survey suggest that surgeons are less likely than urologists and gynecologists to include video in the patient file or to record video to provide information for patients and family. A possible explanation is the fact that both of the latter specialties perform endoscopic procedures such as hysteroscopy and cystoscopy, during which it is customary to include photography or video with the report.

The majority (58.5%) would think it is unlikely they would behave differently during surgery when intra-operative video recording is applied. Even more (82.8%) think it is unlikely that their surgical methods would be altered. An important finding is the fact that residents among respondents find it significantly less unlikely that their behavior or surgical method would be altered (34.2% and 75.4%, respectively). Being in specialist training, it is important for residents to feel at ease and to be able to perform their surgery with as less additional pressure as possible. However, as our previous study has demonstrated, the role of intraoperative video recording in behavioral modification, also known as the “Hawthorne effect”, is negligible [6].

A major concern related to the recording of intraoperative video (and audio) is the risks regarding the privacy of the patient and the operating room personnel alike. This is illustrated by the fact that over a third of respondents find it objectionable to be recognized on intraoperative video recording. Regarding possible medico-legal liability, over half of respondents find it objectionable.

At this moment it is unclear when and for what purposes and by whom these recordings could be accessed. International legal texts mainly focus on the individual’s privacy, and are yet to incorporate specific situations for the surgical setting [22–24].

Overall, the majority of respondents consider the added value of intraoperative video recording for multiple uses. This is less for intraoperative audio recording. The main sentiment in this regard is about significant loss of privacy. For instance, many respondents commented that in the operating room it is of great importance to be able to talk about non-work-related issues for an adequate balance between focus and being at ease. Sometimes, these topics can be of intimate nature. Without the proper delineation of who is able to access such audio recordings, most fear for their privacy and current job satisfaction.

55.7% and 41.5% of respondents recognized the benefit of intraoperative video recording and combined video- and audio recording, respectively, in regard to its supportive role in medicolegal proceedings. In contrary of what is often feared, intraoperative recording could aid in medicolegal proceedings, instead of merely posing risk for medical negligence [25]. The importance of an intraoperative event is often not able to be appraised by an operator during the procedure. Therefore, in this scenario, systematic recording of a procedure in its entirety is necessary, not merely of a selection of procedures or at certain moments when the surgeon “feels like it”.

This survey yielded a response rate of 27.8%, a rate similar to other surveys having approached a comparable number of possible respondents [26]. Also, due to the larger number of invitations, this survey included a high number of replies. With this response rate, however, there is risk for possible imbalance among respondents, e.g. respondents more interested in laparoscopic surgery, in which video recording is already operational, might be more outspoken concerning intraoperative video, compared to respondents of which the majority of procedures are “open surgery” (e.g. transplant surgery, vascular surgery, or trauma surgery).

As the results of this study suggest, the surgical landscape is still divided in terms of intraoperative multimedia recording. Whilst the majority of respondents feel the current method of surgical reporting is insufficient and a large portion are open to the idea of documenting the operative phase on video or audio, there are still certain issues to be sorted out before implementation could be considered. First of all, a significant portion of respondents expressed their concern in regard to potential privacy infringement. Currently no specific law is in effect to shield healthcare providers for their exposure when being recorded during practice. Furthermore, the issue in terms of ownership has not been resolved yet. Up to now, all documentation in healthcare, albeit written, photographed or recorded, are incorporated in the patient file, rendering it patient property by law. In this case, no protection for the healthcare provider is specifically implemented. It is, therefore, imperative that specific legislation will be developed for these methods of intraoperative documentation to adequately protect all subjects in the recordings as well as securing ease of use and harnessing its potential in quality and safety procurement.

In conclusion, the majority of respondents find the current method of operative recording insufficient for future quality requirements. There is support for intraoperative video recording, however, most respondents fear privacy infringement. These concerns are greater for audio recording compared to video recording only. Legislation is necessary before either intraoperative video or audio recording could be implemented to protect not only the patients, but also the healthcare providers.

## Electronic supplementary material

Below is the link to the electronic supplementary material.

Supplementary file1 (DOCX 21 kb)
