# Supplementary material for: Current perspectives on video and audio recording inside the surgical operating room: results of a cross-disciplinary survey
Source: Updates Surg. 2020 Oct 26;73(5):2001–7. doi: 10.1007/s13304-020-00902-7 (PMC8500875; doi:10.1007/s13304-020-00902-7)
Supplement: Supplementary file 1 — Supplementary file1 (DOCX 21 kb) [file 13304_2020_902_MOESM1_ESM.docx]

# Supplementary data – Survey (Translated from Dutch)

## Demographic data

**Question 1.** *What is your current function?*

- Surgeon
- Gynecologist
- Urologist
- Resident in training
- Retired specialist
- Other (specify)

**Question 2A.** *(if surgeon) What is your subspecialization?*

*(Multiple answers possible)*

- Surgical Oncology
- Gastrointestinal Surgery
- Hepatopancreaticobiliary Surgery
- Pediatric Surgery
- Pulmonary Surgery
- Trauma Surgery
- Vascular Surgery
- Not applicable

**Question 2B.** *(if gynecologist) What is your subspecialization?*

*(Multiple answers possible)*

- General Gynecology
- Maternal-Fetal Medicine
- Reproductive Endocrinology and Infertility
- Urogynecology
- Gynecological Oncology
- Not applicable

**Question 2C.** *(if urologist) What is your subspecialization?*

*(Multiple answers possible)*

- General Urology
- Andrological Urology
- Endourology and Stone Disease
- Functional and Reconstructive Urology
- Pediatric Urology
- Not applicable

**Question 2D.** *(if resident in training) For what specialty are you in training?*

- Surgery
- Gynecology
- Urology

**Question 3A.** *(If surgeon, gynecologist or urologist) How many years are you practicing surgery?*

- <5 years
- 5 to 10 years
- 10 to 15 years
- 15 to 20 years
- >20 years

**Question 3B.** *(If resident) What year of the training are you currently in?*

- Year 1
- Year 2
- Year 3
- Year 4
- Year 5
- Year 6

**Question 4.** *What is your workplace?*

- University hospital
- General teaching hospital
- General non-teaching hospital
- Other (specify)

## Current use of operative reporting

**Question 5.** *Do you think that the currently used narrative operative report – without video and/or sound – is sufficient for future quality requirements?*

- Yes
- No

**Question 6.** *As far as you are aware, which techniques are currently used to document surgical procedures in your department?*

*(Multiple answers possible)*

- Endoscopic camera
- External camera recording the surroundings of the operating room
- External camera recording the surgical field (e.g. camera in the OR light)
- Surgical Black Box
- Mobile phone (picture/video/sound)
- Audio recording (microphone)
- Other (specify)
- None of the above

**Question 7.** *Is routine video recording during conventional (‘open’) surgical procedures currently taking place in your department?*

- Yes
- No
- Don’t know

**Question 8.** *Is routine video recording during endoscopic surgical procedures currently taking place in your department?*

- Yes
- No
- Don’t know

**Question 9.** *If surgical procedures are recorded on video in your institution, what is the retention period of these recordings?*

- <30 days
- 30 to 90 days
- 90 days to 1 year
- >1 year
- Don’t know

## Current use of multimedia in the operating room

**Question 10.** *Please indicate of the following actions in what frequency you apply them.*

|  | Never | Rarely | Sometimes | Regularly | Always |
| --- | --- | --- | --- | --- | --- |
| In current practice, do you make video recordings of endoscopic surgical procedures | ⃝ | ⃝ | ⃝ | ⃝ | ⃝ |
| In current practice, do you make video recordings of conventional (‘open’) surgical procedures | ⃝ | ⃝ | ⃝ | ⃝ | ⃝ |

**Question 11.** *If you record your surgical procedures on video, for what purposes?*

*(Multiple answers are possible)*

- Addition to patient file
- For quality control purposes
- For educational purposes
- In the context of proctoring
- To provide information for patients, patients’ family and/or colleagues
- Other

**Question 12.** *Please indicate for the following statements to what extent you agree.*

|  | Very unlikely | Unlikely | Neutral | Likely | Very likely |
| --- | --- | --- | --- | --- | --- |
| I would ***behave*** differently in the operating room when video recording is taking place | ⃝ | ⃝ | ⃝ | ⃝ | ⃝ |
| I would ***perform surgery*** differently in the operating room when video recording is taking place | ⃝ | ⃝ | ⃝ | ⃝ | ⃝ |
| I would ***behave*** differently in the operating room when video ***and*** audio recording is taking place | ⃝ | ⃝ | ⃝ | ⃝ | ⃝ |
| I would ***perform surgery*** differently in the operating room when video ***and*** audio recording is taking place | ⃝ | ⃝ | ⃝ | ⃝ | ⃝ |

**Question 13.** *Please indicate for the following situations, in the context of intraoperative video recording, to what extent you find it objectionable.*

|  | Not at all objectionable unlikely | Not objectionable | Neutral | Objectionable | Very objectionable |
| --- | --- | --- | --- | --- | --- |
| Recognizability of my or my colleague’s identity on the video recordings | ⃝ | ⃝ | ⃝ | ⃝ | ⃝ |
| Potential for medical liability | ⃝ | ⃝ | ⃝ | ⃝ | ⃝ |
| Harmful for the quality of surgical care | ⃝ | ⃝ | ⃝ | ⃝ | ⃝ |

**Question 14.** *Please indicate for the following situations to what extent intraoperative* ***video recording*** *might be of added value.*

|  | Very unlikely | Unlikely | Neutral | Likely | Very likely |
| --- | --- | --- | --- | --- | --- |
| Documenting the operative phase as an addition to the patient file | ⃝ | ⃝ | ⃝ | ⃝ | ⃝ |
| For educational purposes | ⃝ | ⃝ | ⃝ | ⃝ | ⃝ |
| To provide information for patients, family and/or colleagues | ⃝ | ⃝ | ⃝ | ⃝ | ⃝ |
| For quality control purposes | ⃝ | ⃝ | ⃝ | ⃝ | ⃝ |
| In the context of proctoring | ⃝ | ⃝ | ⃝ | ⃝ | ⃝ |
| Supportive evidence in medicolegal proceedings | ⃝ | ⃝ | ⃝ | ⃝ | ⃝ |

**Question 15.** *Please indicate for the following situations to what extent intraoperative* ***video and audio recording*** *might be of added value.*

|  | Very unlikely | Unlikely | Neutral | Likely | Very likely |
| --- | --- | --- | --- | --- | --- |
| Documenting the operative phase as an addition to the patient file | ⃝ | ⃝ | ⃝ | ⃝ | ⃝ |
| For educational purposes | ⃝ | ⃝ | ⃝ | ⃝ | ⃝ |
| To provide information for patients, family and/or colleagues | ⃝ | ⃝ | ⃝ | ⃝ | ⃝ |
| For quality control purposes | ⃝ | ⃝ | ⃝ | ⃝ | ⃝ |
| In the context of proctoring | ⃝ | ⃝ | ⃝ | ⃝ | ⃝ |
| Supportive evidence in medicolegal proceedings | ⃝ | ⃝ | ⃝ | ⃝ | ⃝ |

**Question 16.** *Regarding documentation of surgical procedures, which of the following scenarios would you prefer?*

- Video recordings of the entire surgical procedure
- Video recordings of only the essential steps of the surgical procedure
- Video and audio recordings of the entire surgical procedure
- Video and audio recordings of only the essential steps of the surgical procedure
- No video and audio recordings
